# Effect of fibrin vs cellulose based haemostatic agents with traditional haemostatic procedures in thyroid surgery

**DOI:** 10.12669/pjms.336.13692

**Published:** 2017

**Authors:** Karanikolic Aleksandar, Djordjevic Miodagl, Djordjevic Nebojsa, Golubovic Ilija

**Affiliations:** 1 Dr. Karanikolic Aleksandar, MD, PhD. Medical Faculty Nis, University of Nis, Serbia. Surgical Clinic, Clinical center Nis, Serbia; 2 Dr. Djordjevic Miodag, MD. Medical Faculty Nis, University of Nis, Serbia. Surgical Clinic, Clinical center Nis, Serbia; 3 Prof. Dr. Djordjevic Nebojsa, MD, PhD. Medical Faculty Nis, University of Nis, Serbia. Surgical Clinic, Clinical center Nis, Serbia; 4 Dr. Golubovic Ilija MD. Surgical Clinic, Clinical center Nis, Serbia

**Keywords:** Cellulose-based haemostat, Fibrin-based haemostat, Thyroid surgery

## Abstract

**Objective:**

To compare the efficacy of fibrin-based hemostat and cellulose-based hemostat with traditional procedures in patients undergoing thyroid surgery.

**Methods:**

Between January 2012 and December 2016, 255 patients were scheduled to undergo total thyroidectomy at Surgical Clinic Nis. The patients were randomized to: Group-I use of classic surgical procedures to achieve hemostasis, Group-II use Surgicel and Group-III use Beriplas

**Results:**

A statistically significant reduction of surgical time was found for Group-I compared with Group-II and III (p≤0.01 for both groups). Statistically significant reduction of intraoperative blood loss was found for Group-I compared with Group-II from 102.3mL vs. 86.1mL (p≤0.01) as well as compared to Group-III (mean 77.4mL, p≤0.01). Removal of the wound drain occurred sooner in the Group-II and III compared with Group-I (mean 37.1h vs. 40.1h, p≤0.05; mean 31.2h vs. 40.1h, p≤0.01). There were no significant differences in terms of postoperative morbidity among the groups.

**Conclusions:**

Fibrin based haemostat seems to be an effective hemostatic agent for patients undergoing thyroid surgery. We suggest that, where appropriate, this fibrin-based haemostat should be used as a first line hemostatic agent in thyroidectomy in combination with conventional surgical means of hemostasis.

## INTRODUCTION

Thyroid is one of the best vascularized organs in the human organism, and during the thyroid gland surgery, adequate and accurate hemostasis is very important.^1^,^2^ During thyroidectomy, hemostasis is usually performed using clamp-and-tie maneuvers for the ligation of the thyroid vessels. Other applicable methods include the use of clips and electrocautery and mono/bipolar instruments.

Thompson et al.^3^ standardized extracapsular dissection technique, which led to a significant reduction of morbidity and mortality. Postoperative bleeding in thyroid surgery is rare but can have potentially fatal outcome. Postoperative bleeding occurs in 0.3% to 2% of cases.^4^

Sometimes, the parathyroid glands and laryngeal nerve cannot be seen clearly because of intraoperative bleeding, which increases the risk of their injury as well as the rates of transient and persistent postoperative complications. On the other hand, bleeding can lead to compression and obstruction of the airway, with the occurrence of respiratory distress as a result of laryngopharyngeal edema. The cause of the edema is a difficult venous and lymphatic drainage of the laryngopharyngeal region.^5^

In everyday clinical practice, different local hemostatic agents are used in order to prevent bleeding. The use of these agents is carried out in the course of the surgical procedure when conventional methods of hemostasis cannot stop the bleeding because of the specific location. There are various mechanisms through which local hemostatics can stop the bleeding. Some of them improve primary hemostasis, others stimulate fibrin formation or inhibit fibrinolysis.^6^ Some mechanisms are part of the preparation of a procoagulant substance in combination with a vehicle such as collagen matrix, whereas others use a matrix to provide a template for the endogenous coagulation cascade to achieve hemostasis. Which local hemostatic will be applied during surgery depends on the type of surgery, extent of bleeding, surgeon’s experience and preference, and the product price.

The aim of this study was to compare the efficacy of fibrin-based hemostat (Beriplast®) and cellulose-based hemostat (Surgicel®) with traditional procedures in patients undergoing thyroid surgery, in terms of blood loss, operative time, drainage volume, hospital stay, and postoperative complications.

## METHODS

Between January 2012 and December 2016, 255 patients were scheduled to undergo total thyroidectomy for the thyroid disease at Surgical clinic Nis, Republic of Serbia. To ensure homogeneity between the three treatment groups, we excluded patients with Graves’ disease and other hyperfunctioning thyroid diseases, those with rerosternal goiters, those affected by hemostasis or coagulation disorders, and patients requiring a lymphadenectomy.

The patients were randomly divided into three groups (random sorting through a computerized list of patients), each of them employing a different means of hemostasis: in Group-I, classic surgical procedures were used to achieve hemostasis (ligatures and bipolar electrocauterization alone); in Group-II, oxidized regenerated cellulose patch (Surgicel® cellulose-based hemostat 2.5×5 cm) was applied, whereas in Group-III, the fibrin-based hemostat (Beriplast® P Combi-Set 1ml) was used. In Group-II and III, hemostatic agent should be considered in addition to the classic methods (ligature, bipolar electrocautery). To reduce a potential bias due to interoperator variability, all operations were performed by the same surgical team who had experience in thyroid surgery. All procedures were done under general anesthesia. During surgery, we identified recurrent parathyroid glands and laryngeal nerves.

Serum calcium levels were determined 12 and 24 hours after the surgery. Patients with dysphagia, hoarseness and dyspnea were evaluated with laryngoscopy. On the third postoperative day, patients with uncomplicated postoperative course were discharged if calcium levels were within the normal range (8.5–10.5 mg/dL). All patients were followed up at weeks one, two, and six after the surgery.

The surgeon calculated the operative time. Intraoperative blood loss was calculated by using a graduated suction device and by calculating the amounts of blood collected in the sponges at the end of the surgery. The medical records were reviewed and compared regarding age, sex, diagnosis, estimated intraoperative blood loss, postoperative complications and hospital stay. The drain was removed if the leakage was less than 50 cm3 per 24 hour, and when the secretion changed from the hematic to serous. In our study, drainage is part of the protocol as postoperative blood loss needs to be measured objectively.

The institutional review board approval was obtained. The guidelines of the Declaration of Helsinki on medical protocol and ethics were followed in this study.

Differences among the groups were examined by Fisher Exact Test, Chi square test, one way ANOVA and post hoc Tukey’s multiple comparison tests. Values were expressed as means ± standard deviation (SD). All statistical tests used the 5% significance level. Statistical analyses were performed with statistical software SPSS v.10.

## RESULTS

During the study period, 255 patients underwent total thyroidectomy by the same endocrine surgical team. Hemostasis was achieved using: classic surgical procedures to achieve hemostasis (Group-I, n = 79), oxidized regenerated cellulose patch (Group-II, n = 86) and fibrin-based hemostat (Group-III, n = 87). The three groups were comparable in terms of age, sex, type of diagnosis, and specimen weight (Table-I). No difference was found in age and sex between the groups. There were no differences in the groups regarding thyroid gland weight or diagnosis.

**Table-I:** Patients baseline characteristics.

*Variable*	*(n= 79)*	*(n=86)*	*(n=87)*	*P value*
Mean age, year (range)	45 (20-72)	50 (21-74)	48 (20-73)	NS
*Sex, n (%)*				
Male	26 (32.9)	28 (32.6)	27 (31)	NS
Female	53 (67.1)	58 (67.4)	60 (69)	
*Diagnosis, n (%)*				
Benign thyroid disease	47 (59.5)	50 (58.1)	51 (58.6)	NS
Malign thyroid disease	32 (40.5)	36 (41.9)	36 (41.4)	
Specimen weight, (g)^†^	95.5 (24.3)	89.5 (29.1)	93.1 (23.7)	NS

NS= not statistically significant.

† Values are expressed as means (SD).

**Fig.1:**
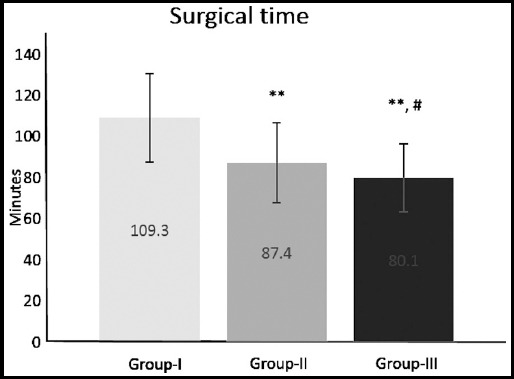
Surgical time in 255 patients undergoing total thyroidectomy for thyroid disease (Group-I – surgical hemostasis, Group-II – Surgicel, Group-III - fibrin-based haemostat). Data are expressed as mean ± SD; ^**^p≤0.01 compared to Group-I; ^#^p≤0.05 compared to Group-II.

Total thyroidectomy was completed in all cases. A statistically significant reduction in the operative time was found for Group-III compared with Group-I and II (Fig.1);^**^p≤0.01 compared with Group-I,^#^p≤0.05 compared with Group-II.

**Fig.2:**
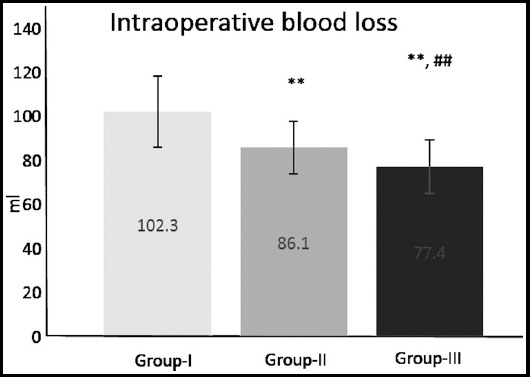
Intraoperative blood loss in 255 patients undergoing total thyroidectomy for thyroid disease (Group-I – surgical hemostasis, Group-II – Surgicel, Group-III - fibrin-based haemostat). Data are expressed as mean ± SD; ^**^p≤0.01 compared to Group-I, ^##^p≤0.01 compared to Group-II.

Also, a statistically significant reduction of intraoperative blood loss was found for Group-III compared with Group-I and II (Fig.2);^**^p≤0.01 compared with Group-I,^##^p≤0.01 compared with Group-II.

**Fig.3:**
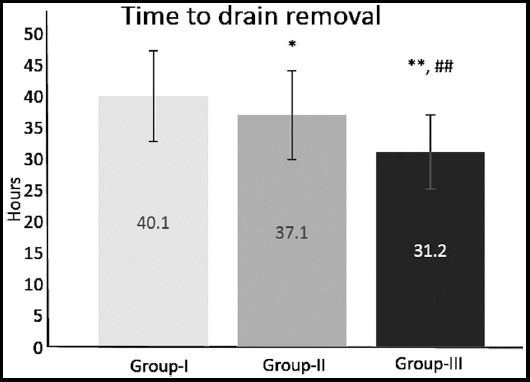
Mean time to removal of postoperative wound drain in 255 patients undergoing total thyroidectomy for thyroid disease (Group-I – surgical hemostasis, Group-II – Surgicel, Group-III - fibrin-based haemostat). Data are expressed as mean ± SD; ^*^p≤0.05 compared to Group-I; ^**^p≤0.01 compared to Group-I; ^##^p≤0.01 compared to Group-II

The removal of the wound drain occurred earlier in Group-III compared to Group-II and Group-I (Fig.3);^*^p≤0.05 compared to Group-I; ^**^p≤0.01 compared to Group-I; ^##^p≤0.01 compared to Group-II.

**Fig.4:**
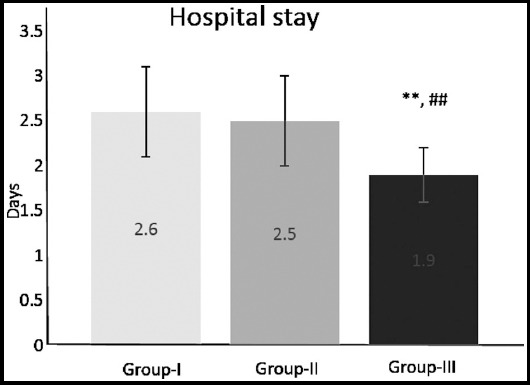
Hospital stay in 255 patients undergoing total thyroidectomy for thyroid disease (Group-I – surgical hemostasis, Group-II – Surgicel, Group-III - fibrin-based haemostat). Data are expressed as mean ± SD; ^**^p≤0.01 compared to Group-I; ^##^p≤0.01 compared to Group-II

There was a statistically significant difference in the length of hospital stay between the groups. Patients in whom fibrin-based hemostat was used (Group-III), had the shortest hospital stay compared to the other two groups of patients (Fig.4);^**^p≤0.01 compared to Group-I; ^##^p≤0.01 compared to Group-II.

There were no significant differences in terms of postoperative morbidity among the groups, as shown in Table-II. Transient hypocalcaemia was 13.9% in Group-I, 7.0% in Group-II, and 6.9% in Group-III. There were no statistically significant differences related to hoarseness and wound seroma between the three groups. There was no permanent complication in the examined groups. Significant postoperative bleeding that lead to reoperation occurred in two cases in Group-I. The bleeding point was located in the superior thyroid artery in one patient and inferior thyroid vein in the other patient. Patients had an uneventful recovery.

**Table-II:** Postoperative morbidity.

*Variable*	*(n=79)*	*(n= 86)*	*(n=87)*	*P value*
Transient hypocalcaemia n (%)	11 (13.9)	6 (7)	6 (6.9)	NS
Wound seroma n (%)	2 (2.5)	1 (1.2)	0 (0)	NS
Hoarseness n (%)	4 (5.1)	2 (2.3)	2 (2.3)	NS
Postoperative hemorrhage, n (%)	2 (2.5)	0 (0)	0 (0)	NS
Permanent complications n (%)	0 (0)	0 (0)	0 (0)	NS
Surgicel granulomata mimicking abscess	0 (0)	1 (1.2)	0(0)	NS

NS = not statistically significant.

In the Surgicel group of patients, six weeks after the surgery, a painful mass was found in one patient, in the paratracheal region on the left side. Computed tomography (CT) showed a heterogeneous soft tissue mass with variable attenuation as well as the areas of calcification. After the surgery, histology showed a conglomerate mass of foreign material with the surrounding inflammatory granuloma. The patient was discharged home on the seventh post-operative day. The patients did not exhibit allergic reactions after the administration of local hemostatic agents.

## DISCUSSION

Thyroidectomy is one of the most common surgical procedures in general surgery. Although this procedure is widely applied, it requires a highly skilled surgical team because of its specificity and complexity. The occurrence of postoperative bleeding is very rare but if it occurs, it can have fatal consequences. In 72% of cases, postoperative hematoma occurs within 6 hours of the surgery, whereas in 89% of cases it occurs within 12 hours of the surgery.^7^The most common signs that indicate there is a postoperative bleeding in association with significant drain tube losses are progressive neck swelling, suture line bleeding, and stridor.

The risk factors related to postoperative bleeding can be put into three groups. The first Group-Includes factors related to the patient (abnormal coagulation status, chronic renal disease, the use of anticoagulation and antiplatelet therapy). The second group of factors is related to the actual thyroid gland disease (Basedow’s disease, toxic multinodular glands, large retrosternal goiter). When it comes to the third group, it reflects on the factors related to the surgical technique itself and the skill of the surgeon.^8^-^10^

Sometimes, light bleeding can occur near the recurrent nerve while dissecting the suspensory ligament (lig. Berry). Precisely for this reason, using methods other than electrocautery and ligatures can lead to achieving the only adequate hemostasis.

Tonante’s study^11^ is the one showing the ability to control the bleeding from retroneural vessels by including usage of collagen and thrombin gelatin granules. To enable achieving an adequate hemostasis while different surgical procedures are being performed, different hemostatic agents and glues are used.

For several decades, cellulose-based hemostats have been used. This hemostatic achieves hemostasis through different mechanisms. Some of them include the following: surface interactions with proteins, blood absorption, platelets, and activation of both the intrinsic and extrinsic pathways.^12^ A valuable hemostatic agent used in surgery because of tissue ability to absorb it, and its inherent hemostatic properties, is Surgicel.^13^ Because of its efficiency and low cost, Surgicel is commonly used in various surgery fields to control bleeding.

Fibrin sealants have been present in surgical practice since the 1970s, with both hemostatic and adhesive properties. Preparations with fibrin sealants have fibrinogen in their composition, which is combined with thrombin, factor XIII and/or an anti-fibrinolytic agent. When the constituent parts are mixed, a cross-linked insoluble fibrin-matrix is formed.^14^,^15^

There are few studies examining the use of local hemostatic agents in thyroid surgery. Testini et al.^16^ compared the effectiveness of FloSeal® matrix hemostatic agent with hemostatic surgical procedures and Tabotamp® in thyroid surgery. In this study, 155 patients were divided into three groups depending on the method of hemostasis that was applied. FloSeal matrix significantly reduces the operative time, as well as the time to removal of wound drain and the length of postoperative stay.

Tartaglia et al.^17^ focused on comparing the efficacy of collagen patch coated with human fibrinogen and human thrombin (CFTP) and oxidized regenerated cellulose gauze versus traditional hemostatic procedures in thyroid surgery. In this study, 226 patients were included. The use of CFTP helped reducing the drainage volume, potentially the bleeding complications, and the hospital stay. Based on these findings, the efficacy of CFTP can be proven, therefore supporting its use in thyroid surgery.

However, not all the studies have shown that routine use of local hemostatic agents is beneficial, comparing to traditional methods of hemostasis in thyroid surgery. A single-blinded, prospective, randomized controlled trial conducted by Amita et al.^18^ measured the efficacy and safety of an oxidized cellulose patch Surgicel with conventional hemostasis in thyroid surgery. The study was conducted on 190 patients.

There were no significant group differences in postoperative wound infection, seroma, hypocalcemia, or recurrent laryngeal nerve palsy. Time to drain removal and length of hospital stay were significantly shorter in the conventional group. A routine use of an oxidized cellulose hemostatic agent has no advantage over conventional hemostasis according to this author.

We found that operative time and intraoperative blood loss were statistically significantly shorter in the fibrin-based hemostat (Beriplast) group of patients than in the other two groups. This difference may be related to the immediate efficacy of the topical hemostatic agent with respect to conventional surgical maneuvers. The removal of the wound drain occurred earlier in the fibrin-based hemostat (Beriplast) group than in the conventional hemostatic surgical procedures group and Surgicel group. Resultantly, the postoperative hospital stay was also significantly shorter in the fibrin-based hemostat (Beriplast) group than in the group of patients undergoing surgical hemostasis or in the Surgicel group.

In terms of morbidity, no differences were found between the groups. Our observations were similar to those of previously published reports.^19^-^21^ This result confirms the findings that morbidity secondary to thyroid surgery largely depends on the surgical skill, and not so much on the kind of intraoperative procedure used to achieve hemostasis.

In our study, the overall incidence of postoperative hemorrhage was 2.50% (two out of 255 cases) with postoperative hematoma appearing 8h and 15h after the surgery. The postoperative bleeding occurred in the group where hemostasis was achieved using the classic surgical procedures.

The incidence of permanent complications after total thyroidectomy varies widely. However, in experienced hands, this incidence is low. Clark reported no permanent nerve palsy and a 1% incidence of permanent hypoparathyroidism in 82 consecutive patients.^22^ Similarly, Mishra et al.^23^ reported an incidence of 0.8% nerve injury and 1.6% hypoparathyroidism. This study showed that there were no significant differences in terms of postoperative complications among the three groups.

In the Surgicel group of patients, six weeks after the surgery, in one patient we found a painful mass in the paratracheal region on the left side. Although Surgicel is a relatively non-irritant substance and is completely absorbed by the body in most instances, it is a foreign body and should be used as rarely as possible. Surgeons and radiologists should be familiar with the appearance of retained Surgicel in X-rays,^24^ sonograms^25^ and computed tomography scans.^26^

## CONCLUSION

In our initial experience, Beriplast seemed to be an effective hemostatic agent for patients undergoing thyroid surgery. In patients with Beriplast type of hemostasis, we found that operative time, intraoperative blood loss, hospital stay and the time to removal of wound drain were significantly reduced. These findings confirm the efficacy of Beriplast, promoting its use in thyroid surgery.

### Authors’ Contribution

**KA** conceived, designed and did statistical analysis & editing of manuscript.

**DM, GI** did data collection and manuscript writing.

**DN** did review and final approval of manuscript.
